# The Impact of a *Preventive Dentistry Programme* on Oral Health: A Pilot Study

**Published:** 2017-12

**Authors:** Anna NADAŽDYOVÁ, Dagmara SIROTŇAKOVÁ, Martin SAMOHÝL

**Affiliations:** 1. Dept. of Stomatology and Maxillofacial Surgery, Faculty of Medicine, Comenius University in Bratislava, Bratislava, Slovakia; 2. Private Dental Clinic, Bratislava, Slovak Republic; 3. Institute of Hygiene, Faculty of Medicine, Comenius University in Bratislava, Bratislava, Slovakia

## Dear Editor-in-Chief

Dental caries is considered to be a major public health problem (1,2). The Decayed, Missing, Filled (DMF) index is well established as the key measurement of the prevalence of caries in oral health evaluation (3,4). The DMF index has been recommended by the WHO as a parameter by which to assess the dental health situation in various societies (5). Dental surgeons working in the public sector have an important role to play in school dental health programmes (6). Oral diseases are ubiquitous and they carry not only physical but also socioeconomic and psychological consequences for the patients (7).

This study aimed to analyze the impact of a *preventive dentistry programme* on oral health.

The study group consisted of subjects (n=45; 37.8% male; mean age 5.1±0.8 yr) who regularly participated in a preventive dental programme educating children on good oral hygiene and healthy eating habits. The control group consisted of patients who did not participate in any preventive dental programme (n=25; 44.0% of males; mean age 6.0±0.0 yr). The *children’s oral health* was evaluated using the DMF index.

$$\mathrm{DMF}=\frac{D+M+F}{32}*100$$

*D* represents the number of decayed teeth, *M* represents the number of missing teeth and ***F*** represents the number of filled teeth.

A statistically significantly higher DMF index was observed in the control group (non-participation in the preventive dental programme) than in the study group. A significant difference in females (*P*=0.014) and the 5–6 yr age group (*P*=0.004) between the study group and the control group was found (Table 1).

**Table 1:** Mean DMF index scores according to gender and age (n=70)

***Variables***	***Study group (n = 45)***			***Control group (n = 25)***				P*^3^*
**Category**	**Subcategories**	**n (%)**	**DMF index x (SD)**	***P***^1^	**n (%)**	**DMF index x (SD)**	***P*^2^**	
Gender	Male	17 (37.8)	8.3 ± 10.1	0.766	25 (50.0)	18.6 ± 12.5	0.060	0.0
	Female	28 (62.2)	9.3 ± 11.8				0.014	02
Age (yr)	Mean (x ± SD)		5.1 ± 0.8			6.0 ± 0.0		
	3 – 4	6 (13.3)	7.3 ± 8.5	0.665	25 (50.0)	18.6 ± 12.5	-^*^	0.0
	5 – 6	39 (86.7)	9.1 ± 11.5				0.004	02

* No subjects were in the control group (age group 3 – 4 y.). We were not able to detect a significant difference.

1 significant difference in study group//

2 significant differences in subcategories between study group and control group

3 significant differences in categories between study group and control group

This preventive dental programme is one factor that can lead to good oral hygiene and the prevention of tooth caries in children.

The significance of this preventive dental programme was confirmed in our study, where a significantly lower DMF index was observed in subjects who regularly participated in the preventive dental programme than in children who did not participate in the preventive dental programme. Besides the role of the parents, another important role, which affected the children’s outcomes (where the preventive dental programme took place), was the role of the teachers. A disadvantage in this preventive dental programme was the absence of some children due to their parents’ refusal to allow them to participate in the programme. Parents play an important role in preventing dental caries in children not only in terms of individual prevention but also because parents accompany the child to the dentist’s chair during professional preventive appointments.
